# Removal of helminth eggs by centralized and decentralized wastewater treatment plants in South Africa and Lesotho: health implications for direct and indirect exposure to the effluents

**DOI:** 10.1007/s11356-018-1503-7

**Published:** 2018-02-24

**Authors:** Isaac Dennis Amoah, Poovendhree Reddy, Razak Seidu, Thor Axel Stenström

**Affiliations:** 1 Institute for Water and Wastewater Technology, Durban University of Technology, PO Box 1334, Durban, 4000 South Africa; 2 Department of Community Health Studies, Faculty of Health Sciences, Durban University of Technology, PO Box 1334, Durban, 4000 South Africa; 3 Water and Environmental Engineering Group, Institute for Marine Operations and Civil Engineering, Norwegian University of Science and Technology, Ålesund, Norway

**Keywords:** Wastewater treatment, South Africa, Lesotho, Wastewater irrigation, Soil-transmitted helminths, QMRA

## Abstract

Wastewater may contain contaminants harmful to human health; hence, there is the need for treatment before discharge. Centralized wastewater treatment systems are the favored treatment options globally, but these are not necessarily superior in reduction of pathogens as compared to decentralized wastewater treatment systems (collectively called DEWATS). This study was therefore undertaken to assess the soil-transmitted helminth (STH) and *Taenia* sp. egg reduction efficiency of selected anaerobic baffled reactors and planted gravel filters compared to centralized wastewater treatment plants in South Africa and Lesotho. The risk of ascariasis with exposure to effluents from the centralized wastewater treatment plants was also assessed using the quantitative microbial risk assessment (QMRA) approach. Eggs of *Ascaris* spp., hookworm, *Trichuris* spp., *Taenia* spp., and *Toxocara* spp. were commonly detected in the untreated wastewater. The DEWATS plants removed between 95 and 100% of the STH and *Taenia* sp. eggs, with centralized plants removing between 67 and 100%. Helminth egg concentrations in the final effluents from the centralized wastewater treatment plants were consistently higher than those in the WHO recommended guideline (≤ 1 helminth egg/L) for agricultural use resulting in higher risk of ascariasis. Therefore, in conclusion, DEWATS plants may be more efficient in reducing the concentration of helminth eggs in wastewater, resulting in lower risks of STH infections upon exposure.

## Introduction

Municipal wastewater contains a variety of pathogens, reflecting the carrier state and infection levels in the community (Carr et al. 2011; Hanjra et al. 2012). The contamination of surface water with untreated or partially treated wastewater may as a consequence lead to adverse health implications (Ahmed et al. 2016; Petterson et al. 2016). There is an epidemiological link between gastro-intestinal diseases and contact with fecally contaminated surface water (Thurston et al. 2001; Amoah et al. 2016). Treatment of wastewater before discharge into surface water bodies will therefore function as a barrier; efficiency of treatment however differs impacting on the reduction of risks achievable (Hussain et al. 2001; Hussain et al. 2002; Qadir et al. 2015). Centralized wastewater treatment plants (WWTPs) are the main wastewater treatment option globally, especially in developed countries. The major bottleneck in the establishment of these centralized WWTPs is the exorbitant costs associated with their construction, operation, maintenance (Massoud et al. 2009), and cost of transportation of the wastewater (UN-Water 2015). According to the UN World Water Development Report 2015, these costs could be reduced considerably by treating wastewater close to the source using simple technologies. The issue of costs of constructing and operating of wastewater treatment plants is mainly a challenge in poor settings (Massoud et al. 2009); access to finance for these investments therefore acts as the main stumbling block (Hanjra et al. 2015; Duchin 2016).

Some of the widely used decentralized wastewater treatment technologies are constructed wetlands, anaerobic baffled reactors (ABRs), upflow anaerobic sludge blankets (UASBs), waste stabilization ponds, aerated lagoons, and oxidation ditches (Elmitwalli et al. 2002; Istenic et al. 2014; Masi et al. 2015). The use of ABRs has increased over the last 10 years due to their low maintenance requirements, simple and inexpensive construction, and stable operational conditions (Tilley et al. 2014; Reynaud and Buckley 2016). Although decentralized wastewater treatment plants, such as the ABRs, have the potential to eliminate some of the challenges associated with centralized wastewater treatment, there is limited information on the achievable pathogen reduction, especially the soil-transmitted helminths (STHs) and other helminths (von Sperling et al. 2003; Foxon et al. 2004; Nasr et al. 2009). In fact, STHs are recognized as a major public health problem affecting over 1.5 billion people worldwide (WHO 2015), with *Ascaris* spp., hookworm, and *Trichuris* spp. infections the most common (Pullan et al. 2014). These infections are associated with low-income countries, mainly occurring in Sub-Saharan Africa, Asia, and South America (WHO 2015).

The increasing reuse of wastewater is making it very important to determine the concentration of these pathogens in effluents from ABR systems. An essential public health concern with wastewater reuse is the health treat from STH infections (WHO 2006) especially in endemic regions. The WHO guidelines for wastewater reuse proposed a guideline value of < 1 helminth egg/L for wastewater intended for unrestricted agriculture, aimed at reducing risks of infections (WHO 2006). Reuse of wastewater and sludge has been associated with elevated STH infections globally (Fuhrimann et al. 2014; Fuhrimann et al. 2016; Contreras et al. 2017; Gyawali 2017). In South Africa, Gumbo et al. (2010) reported a higher prevalence of hookworm infections among farmers using wastewater for irrigation. STHs are a major health concern due to their long periods of persistence in the environment, from a few months up to years (Bethony et al. 2006).

In this study, the STH and *Taenia* spp. egg reduction efficiency of selected centralized and decentralized (ABR coupled with planted gravel filters (PGFs)) plants in South Africa and Lesotho was assessed and compared. In addition, a comparison of the risk of *Ascaris* spp. (as a surrogate for STHs) infections for different exposed populations was estimated to provide a public health perspective to the choice of treatment approach, especially within the context of water reuse.

## Material and methods

### Study area and sampling points

#### Centralized wastewater treatment plants

Wastewater samples were taken from three (3) centralized wastewater treatment plants (WWTPs), all within the eThekwini Municipality of KwaZulu-Natal province in South Africa. This municipality is known globally for its achievements in the field of water and sanitation; therefore, the results from this study will add to the efforts of the municipality in the provision of safe sanitation. The treatment steps within these WWTPs are similar, with the main stages being mechanical grit removal trap, flow division chamber, raw sewage pump station, reaction tank/biological reactor/biological filters, clarifiers, chlorine contact tank, and chemical dosing facilities. Table 1 summarizes the characteristics of the centralized WWTPs studied.

**Table 1:** Characteristics of centralized wastewater treatment plants studied

Plant	Capacity (mL/day)	Size of population served	Characteristics of population served	Sampling points
WWTP A	10.98	30,200	Low- and middle-income individuals	Influent, outlet of rotating biological filters (RBF), outlet of settling tank and outflow of the maturation ponds
WWTP B	4.69	13,800	High- and middle-income households	Influent, outlet of clarifier and effluent (after chlorination)
WWTP C	10.08	29,800	Middle-income households	Influent, outlet of clarifiers, outlet of the chlorination tanks, and outflow of the maturation ponds

#### Decentralized wastewater treatment plants

The decentralized treatment plants are ABRs, with planted gravel filters (PGFs) for final treatment and collectively referred to as decentralized wastewater treatment system (DEWATS). The South African DEWATS plant is at an experimental site in Durban, designed to treat domestic wastewater from about 80 households, with a design capacity for a total of 462 persons. The DEWATS plant is part of a research site managed by the eThekwini municipality; this is aimed at studying the performance of DEWATS systems in treating domestic wastewater under different hydraulic conditions. This plant has an initial two-chamber settling step (also serving as a biogas collection point), and from this, the wastewater is distributed into three parallel ABR treatment trains. Two chambers of anaerobic filters (AFs) follow each ABR train. The final polishing steps are planted gravel filters (PGFs), both vertical and horizontal. Samples were taken from the inlet, after the AFs and finally after the PGFs.

#### Sampling

Approximately five (5) liters of wastewater was taken from each sampling point within the WWTPs studied. Composite samples (in triplicates) were taken based on consecutive subsamples at intervals till the required volume is reached. For a five (5)-liter sample, samples were taken ten times in approximate volumes of 500 mL each.

Sampling at the centralized WWTPs and the DEWATS plant in Durban was done monthly from January to October 2016, and sampling of the DEWATS plants in Lesotho was in June 2015 (five plants) and August 2016 (ten plants). Ten samples were taken from each treatment step for each of the plants to account for variability.

### Laboratory analysis

Sample analysis for the STH eggs was carried out using a new revised methodology (developed in our laboratory) based on the principles of sedimentation and flotation. Briefly, samples were poured through a 100-μm sieve onto a 20-μm sieve (Wirsam Scientific ad Precision Equipment (Pty) Ltd). The contents on the 20-μm sieve were carefully washed into 50-mL centrifuge tubes and centrifuged for 10 min at 3000 rpm. The supernatants were discarded and ZnSO_4_ solution (specific gravity of 1.30) added to a total volume of 50 mL. After resuspension, the mixture was then centrifuged again at 2000 rpm for 10 min. The supernatant was poured through the 20-μm sieve, and the contents of the sieve washed under running water into a 50-mL centrifuge tube and centrifuged at 3000 rpm for 10 min. Supernatants were discarded and the pellets incubated in 0.1 N sulfuric acid for 28 days. The pellets were re-suspended after incubation screened under the microscope at × 100 magnification and further examined at × 400 to determine the stage of development (necessary for the determination of potential viability). Only potentially viable eggs, based on morphology and presence of motile larvae, were counted.

### Statistical analysis

Descriptive analysis to assess the mean concentration and distribution of eggs in the samples was performed using GraphPadPrism version 7.0 (GraphPad Software Inc). Analysis of variance as well as *t* test was performed to determine the statistical difference between the concentrations of the STH eggs and removal efficiencies between/within the WWTPs at 95% confidence interval*.* Probability distribution functions (PDFs) were fitted to the concentration of STH eggs detected in the different samples analyzed using @Risk version 4.5.2 professional edition (Palisade Corporation) added on to Microsoft Excel. The best PDF that described the data was determined by assessing the Akaike information criteria (AIC). The STH removal efficiencies were calculated using the following formula:

$$ \%\mathrm{Eff}=\kern0.75em \frac{Cinf- Ceff}{Cinf}\times 100 $$

where “*Cinf*” is the concentration of eggs in the influent and “Ceff” is the concentration of eggs in the effluent of the respective plants.

### Assessment of risk of *Ascaris* sp. infection

The quantitative microbial risk assessment (QMRA) approach was used to estimate the infection risks associated with direct and indirect exposure to effluents from the centralized WWTPs. This was performed for only the centralized WWTPs due to limited data for accurate assessment of risks for the DEWATS systems. The approach involved the interlink steps of the following: (a) hazard identification, (b) exposure assessment, (c) dose-response assessment, and (d) risk characterization (Haas et al. 2014).

#### Hazard identification

*Ascaris* spp. was chosen as the main organism for the assessment of risk of infections associated with exposure to the effluents. Several studies have shown a significant relationship between direct/indirect exposure to wastewater (e.g., wastewater irrigation and consumption of wastewater irrigated vegetables) and STH infections (especially ascariasis) (Navarro and Jimenez 2011; Amoah et al. 2016; Amoah et al. 2018). *Ascaris* spp. eggs can survive for long periods of time under adverse environmental conditions (Feachem et al. 1983) and has therefore been suggested as the index organism for QMRAs in developing countries by the WHO (2006). In addition, *Ascaris* spp. is the only STH with a dose-response model.

#### Exposure assessment

Exposure assessment involves the determination of the “number of organisms that correspond to a single exposure (termed the dose) or the total number of *Ascaris* spp. eggs that will constitute a set of exposures” (Haas et al. 2014). In this study, two main exposure groups were assessed, namely, (a) occupational exposure and (b) community exposure.

##### Occupational exposure scenario

Irrigation of crops especially vegetables, on small scale/household level, could expose the farmers to *Ascaris* spp. eggs in the irrigation water (treated wastewater). The risk of infection for the farmers using the effluent from these WWTPs for irrigation was therefore quantified and compared between the WWTPs. In this study, the volume ingested was assumed to be uniformly distributed from 1 to 5 mL per irrigation event (WHO 2006). The dose of *Ascaris* spp. eggs ingested by the farmers per day (“*λ*”) was therefore determined using the following formula:

$$ \lambda ={C}_{\mathrm{raw}}\times V $$

where “*C*_raw_” is the concentration of *Ascaris* spp. eggs per milliliter of the final effluents (irrigation water) and “*V*” is the volume (mL/day) of water accidentally ingested by farmers. Frequency of exposure was also assumed to be uniformly distributed from 120 to 140 days per year based on information from farmers in the study area.

##### Community exposure scenario

Community exposure to the final effluents from the investigated WWTPs could also lead to STH infections through the following exposure routes:

1. Recreational/accidental exposure to the effluents: Exposure to the final effluents either intentionally or unintentionally was considered assuming eggs are in the infective stage. Immersion in the maturation ponds (in the case of some of the centralized WWTP) was the main exposure scenario. In some of the WWTPs, the maturation ponds are not fenced and therefore accessible by the general community. In addition, the final effluents are discharged into surface water bodies that run through communities where exposure might occur. In this instance, it is assumed that the concentration of the STH eggs remains constant irrespective of dilution or egg die-off (assuming a worst case scenario). The different exposure scenarios and the volume of water ingested are presented in Table 2.
2. Consumption of wastewater irrigated vegetables: The risk of STH infection for consumers of crops irrigated with effluents from the WWTPs was modeled using lettuce as a surrogate for all vegetables. The dose (λ) of *Ascaris* spp. eggs (λ; no. ingested per person per day) resulting from consumption of the effluent irrigated lettuce was modeled as follows:

**Table 2:** Points of exposure with assumptions based on volume ingested and frequency of exposure

Exposure scenario/assumptions for dosage	Volume ingested (mL or g)	Frequency	Reference
(Un)intentional immersion/swimming at maturation ponds or effluent contaminated surface water	Uniform distribution (10, 15)	Uniform distribution (64,128)*	Dorevitch et al. 2011
Volume caught on lettuce	Normal distribution (0.108, 0.019)		Hamilton et al. (2006)
Daily per capita intake of vegetables	Pert distribution (25, 50, 75)	Uniform distribution (156,160)*	Sant’Ana et al. 2014

*Assumption

$$ \lambda = VIc $$

where “*V*” is the volume of effluent (irrigation water) caught on the surface of the lettuce plant following irrigation (mL g^−1^), “*I*” is the mean per capita intake of lettuce (g person^−1^ day^−1^), and “*c*” is the concentration of *Ascaris* spp. eggs in the final effluents being used for irrigation (no. mL^−1^). There is a large variation on the volume of irrigation water caught on the surface of vegetables following irrigation, and for this study, this volume was assumed to be normally distributed as reported by Hamilton et al. (2006). In addition, it was assumed that there would not be any reduction in the concentration of these eggs either through natural die-off or washing.

#### Dose-response assessment

The *Ascaris* spp. infection risk associated with the different exposure pathways was assessed using the exponential dose-response model (Westrell 2004, Seidu et al. 2008), which is given as follows:

$$ {P}_{\mathrm{inf}}=1-{e}^{- rd} $$

where *P*_inf_ is the *Ascaris* infection risk associated with the ingestion of *d* number of infectious *Ascaris* spp. and *r* is a dimensionless infectivity constant. In this study, *r* value of 0.039 was used (Navarro et al. 2008). The dose of *Ascaris* spp. egg per exposure scenario was modeled by fitting probability distribution functions (PDFs) to the concentration of these eggs as determined in this study. Table 8 in the Appendix describes the various PDFs that best described the *Ascaris* spp. egg concentrations in the effluents from the WWTPs.

#### Risk characterization

In the risk characterization, all the outcomes of the hazard identification, exposure assessment, and dose-response assessment were combined to characterize the severity of *Ascaris* spp. infection. The annual infection risk (*P*_A_) associated with multiple exposures was determined using the following formula:

$$ {P}_{\mathrm{A}}=1-{\left(1-{P}_{\mathrm{inf}}\right)}^n $$

where *P*_inf_ is the risk of infection from a single exposure to a dose *d* of *Ascaris* spp. and *n* being the number of days of exposure to the single dose *d* (Sakaji and Funamizu 1998). For the scenario of farmers’ ingesting both irrigation water and crops, the combined annual risk of infection was determined by using the following formula:

$$ {\pi}_{\mathrm{t}}=1-\left(1-{\pi}_{\mathrm{i}}\right)\left(1-{\pi}_{\mathrm{x}}\right) $$

where “*π*_t_” is the combined annual risk of infection from exposures to irrigation water and crops, “*π*_i_” is the *Ascaris* spp. infection risk resulting from accidental ingestion of irrigation water, and “*π*_x_” is the *Ascaris* spp. infection risk resulting from consumption of wastewater irrigated crops (Haas et al. 2014). All risk models were subjected to Monte Carlo simulations of 10,000 iterations for probability of infections. These models were constructed in Microsoft Excel using the @Risk 7.5 (Palisade Corporation) software add-on to Excel.

## Results

### Occurrence and removal of STHs and *Taenia* spp. in centralized WWTPs and DEWATS

#### Concentration of STH and *Taenia* sp. eggs in raw wastewater at the centralized WWTPs

Different species of STHs at varying concentrations were detected in the influent of the centralized WWTPs (Table 3). *Ascaris* spp. was the most prevalent STH detected, with concentrations ranging from 0 to 201 eggs/L (Table 3). Samples from WWTP A had higher concentrations of almost all the STH eggs (except for *Trichuris* spp.) than the other two WWTPs. Concentration of *Ascaris* spp. eggs did not vary statistically between the WWTP A and WWTP B; same trend was observed for the non-STH, *Taenia* spp. (Table 3). However, concentrations of *Ascaris* spp. and hookworm varied significantly between WWTP B and WWTP C.

**Table 3:** Concentration of STH and *Taenia* spp. eggs in the influent and effluent of the wastewater treatment plants studied

	WWTP A		WWTP B		WWTP C		DEWATS—Durban		DEWATS—Lesotho	
	Influent	Effluent	Influent	Effluent	Influent	Effluent	Influent	Effluent	Influent	Effluent
*Ascaris* spp.*	91 (± 101.5)	2.2 (± 8.4)	16 (± 24.8)	2.4 (± 8.0)	55 (± 45.2)	3.8 (± 2.6)	0.4 (± 0.9)	N/D	87 (± 96)	2.3 (± 1.5)
Hookworm	61 (± 52.1)	3.8 (± 12.2)	15 (± 16.2)	2.5 (± 5.2)	18 (± 18.5)	2.6 (± 4.5)	13 (23.4)	N/D	26 (± 32)	0.19 (± 0.19)
*Trichuris* spp.*	16 (± 12.2)	1.6 (± 8.0)	4.6 (± 1.2)	1.2 (± 1.0)	23 (± 21.7)	13 (± 6.0)	N/D	N/D	12 (± 8)	0.25 (± 0.25)
*Taenia* spp	29.6 (± 9.8)	8.4 (± 8.0)	6.4 (± 2.4)	1.4 (± 2.1)	9.8 (± 8.8)	3.2 (± 8.0)	N/D	N/D	2.3 (± 2.4)	0.25 (± 0.17)
*Toxocara* spp	14 (± 20.1)	1.3 (± 3.1)	7.8 (± 6.1)	1.3 (± 2.2)	9.2 (± 8.9)	3.0 (± 8.0)	N/D	N/D	N/D	N/D

*Significant difference in egg concentrations (*p* < 0.05)

**Fig. 1:**
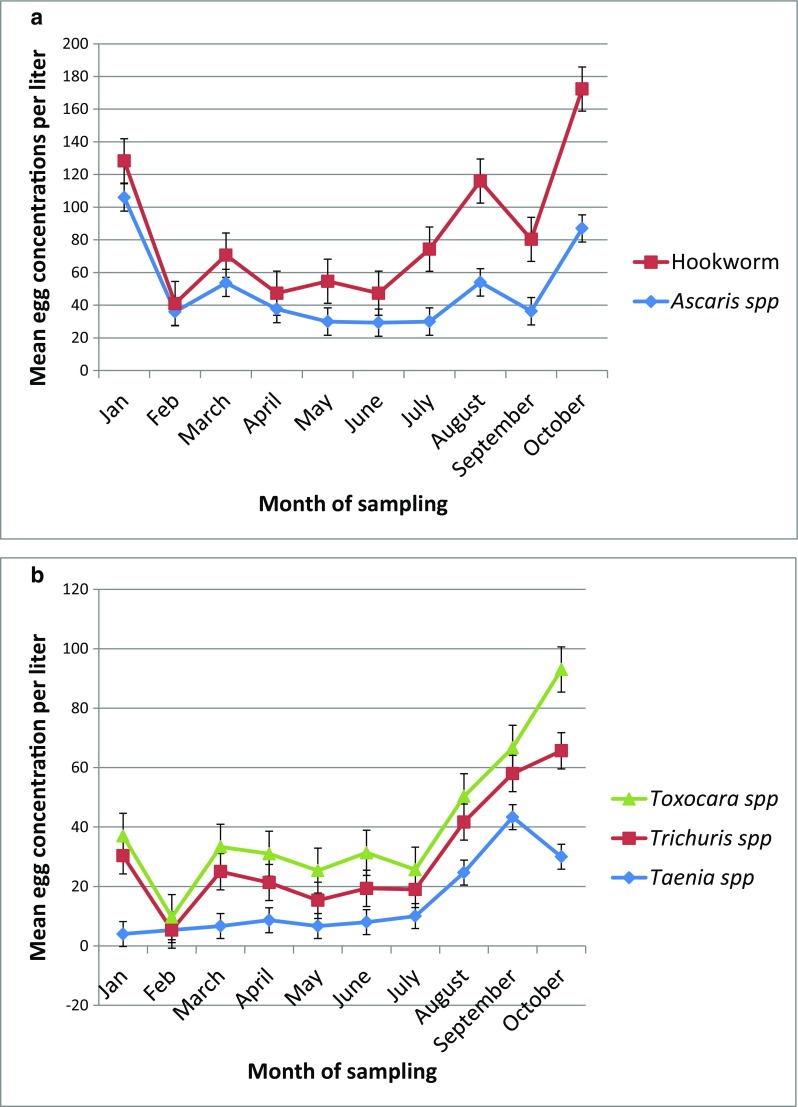
**a** Variation in mean *Ascaris* spp. and hookworm egg concentrations in the raw wastewater at the centralized WWTPs over the study period (*n* = 10). **b** Variation in mean *Toxocara* spp., *Trichuris* spp., and *Taenia* spp. concentration in the raw wastewater at the centralized WWTPs over the study period (*n* = 10)

Variation in the mean concentration of eggs was recorded for the various months throughout the study. Irrespective of the WWTP, these variations followed a similar trend with no significant difference between the WWTPs (*p* value > 0.05) at a said month. Therefore, the concentrations were combined and the mean values are presented in Fig. 1a, b. *Ascaris* spp. and hookworm eggs recorded high concentrations in January and October (Fig. 1a), with the other STH and *Taenia* spp. eggs peaking in January and again steadily from July to October. However, *Taenia* spp. egg concentrations reduced in October (Fig. 1b).

#### Concentration of STH and *Taenia* spp. eggs in raw wastewater at the DEWATS treatment plants

Raw wastewater at the DEWATS plant in Durban only contained eggs of *Ascaris* spp. and hookworm. These were only detected during one (September) month throughout the 10-month study period, in relatively low concentrations. These as well as the corresponding concentrations for the Lesotho treatment plants, for the occurrence of *Ascaris* spp., hookworm, *Taenia* spp., and *Trichuris* spp., are given in Table 3. During the first sampling in June 2015, in Lesotho, only two out of the five DEWATS plants contained *Ascaris* spp. and hookworm eggs in the raw wastewater. In the second sampling in August 2016, additional plants were included with STH eggs occurring more in the raw wastewater. A direct comparison of mean concentrations in the original five (5) DEWATS plants did not give any statistical differences (*p* value ≥ 0.05) between the two sampling rounds. *Ascaris* spp. egg concentrations varied significantly between the raw wastewaters from Durban and Lesotho, except for hookworm concentrations that did not show any statistical significant difference (Table 3). STH egg concentrations varied between the individual DEWATS plants in Lesotho, but the differences were not statistically significant here either.

#### Concentration of STH and *Taenia* spp. eggs in effluents from centralized wastewater treatment plants

All helminth species detected in the raw wastewater (“Concentration of STH and *Taenia* spp. eggs in raw wastewater at the centralized WWTPs”) were recorded in the final effluents of the centralized WWTPs. However, the concentrations varied between the plants, exemplified by *Ascaris* spp. with the highest values in effluents from WWTP C (3.8 (± 2.6) eggs/L), while effluents from WWTP A contained the highest concentrations of both hookworm (3.8 (± 12.2) eggs/L) and *Taenia* spp. (8.4 (± 8.0) eggs/L). Although there were differences in the concentration of STH eggs in the final effluents between the WWTPs, these were not significant except for *Trichuris* spp. concentrations (*p* value ≤ 0.05) (Table 4). Within the individual WWTPs there was variation in the concentrations of the various STH eggs detected (Table 3). The difference in the STH egg concentrations between the influent and effluent was statistically significant (*p* value ≤ 0.05).

**Table 4:** Mean percentage (± SD) removal of STH and *Taenia* spp. eggs from the wastewater treatment plants studied

	*Ascaris* spp.		Hookworm		*Trichuris* spp.		*Taenia* spp.		*Toxocara* spp.	
	Mean (±SD)	Range	Mean (± SD)	Range	Mean (± SD)	Range	Mean (± SD)	Range	Mean (± SD)	Range
WWTP A	96 (± 1.8)	88–100	86 (± 6.4)	33–100	80 (± 9.9)	20–100	96 (± 4.4)	60–100	89 (± 6.1)	40–100
WWTP B	89 (± 4.4)	67–100	72 (± 12)	0.0–100	96 (± 3.7)	67–100	82 (± 8.8)	20–100	82 (± 9.6)	25–100
WWTP C	90 (± 3.5)	67–100	83 (± 8.7)	20–100	56 (± 11)	0.0–100	73 (± 10)	25–100	71 (± 11)	20–100
DEWATS Lesotho	99 (± 0.35)	95–100	100 (± 0.29)	95–100	98 (± 2.1)	67–100	100 (± 0.15)	98–100	–	–

*SD* standard deviation

#### Concentration of STH eggs in effluents from the DEWATS plants

There were no STH eggs detected in the final effluents from the DEWATS plant in Durban. However in Lesotho, eggs of all the STH groups, except *Toxocara* spp., which occurred in the untreated wastewater, were found in low numbers, in the effluents (Table 3). For instance, the highest STH egg found was *Ascaris* spp. (2.3 (± 1.5) eggs/L). These concentrations varied between the various DEWATS plants in Lesotho, but during June 2015 sampling, there was no STH egg in the final effluents. The mean concentrations in the final effluents (Table 3) are from the ten DEWATS plants sampled in August 2016. There was no statistically significant variation in the STH egg concentrations in the final effluents from the individual DEWATS plants that had positive samples in the second sampling.

#### STH and *Taenia* sp. egg removal efficiency of the wastewater treatment plants

The overall removal efficiency for the various centralized WWTPs and DEWATS varied greatly, with difference in the removal achieved for the different STHs and *Taenia* spp. as well. WWTP A had mean removal percentages from 80 (± 9.9) % to 96 (± 1.8) %, 72 (± 12.0) % to 96 (± 3.7) % for WWTP B, and 56 (± 8.7) % to 90 (± 3.5) % for WWTP C. The DEWATS in Lesotho recorded removal efficiencies from 98 (± 2.1) % to 100 (± 0.29) %; a complete removal of STH and *Taenia* spp. eggs in the DEWATS plant in Durban was recorded.

The percentage of the individual helminth eggs removed varied; however, *Ascaris* spp. egg removal was consistently high irrespective of the treatment plant. For instance in WWTP A and C, removal of *Ascaris* spp. eggs was the highest (96 (± 1.8) % and 90 (3.5) % respectively). In WWTP B, removal of *Trichuris* spp. eggs was highest (96 (± 3.7) %) (Table 4). Within the DEWATS plants with positive samples, the removal percentages varied. Plants with accumulation of biogas within the treatment system reported significantly lower helminth egg removals. However, the DEWATS plants achieved a consistently higher STH and *Taenia* spp. egg removal than the centralized WWTPs (Table 4), with removal of *Ascaris* spp. being the highest (99 (± 0.35) %).

The efficiency of the various WWTPs in removing STH and *Taenia* spp. eggs varied within the WWTPs depending on the treatment step. In WWTP A, the highest egg removal occurred in the maturation ponds for *Ascaris* spp. (86 (± 19) %). The settling tanks (both primary and secondary) also contributed to the removal of the STH and *Taenia* spp. eggs, with 44 (± 38) % and 51 (± 44) % removal of *Taenia* spp. and *Trichuris* spp. respectively in the primary settling tanks. The secondary settling tanks removed 44 (± 38) % and 51 (± 44) % of hookworm and *Toxocara* spp. eggs respectively. For WWTP B, the highest reduction was achieved during the clarifier stage with 50 (± 40) % of hookworm eggs. Additionally, *Ascaris* spp. and *Taenia* spp. eggs were best removed at the post-clarifier stage, with 48 (± 47) % and 30 (± 48) % removal respectively. STH egg removal in WWTP C maturation ponds ranged from 44 (± 43) % for hookworm to 53 (± 50) % for *Toxocara* spp. The secondary settling tanks also resulted in an additional egg removal, with 50 (± 29) % for *Ascaris* spp. eggs, 57 (± 36) % for hookworm and 63 (± 34) % for *Trichuris* spp.

In the DEWATS plants, the highest reduction was achieved during the anaerobic treatment step (ABR section), with removals ranging between 72 (± 24) % (*Taenia* spp) to 90 (± 38) % (*Ascaris* spp).

### Quantitative risk assessment according to exposure scenarios

#### Probability of *Ascaris* sp. infection for farmers using treated wastewater for irrigation (occupational exposure)

Reuse of the effluents from the centralized WWTPs for irrigation poses risks of *Ascaris* sp. infections, with effluents from WWTP C giving the highest mean risks of 4.8 × 10^−4^ (± 9.9 × 10^−6^). The variation in infection risk from one-time exposure during irrigation was found to be statistically insignificant (*p* ≤ 0.005) (Table 5). Multiple/annual exposure/s to the effluents would result in increased risks of infections (Table 5), using the assumptions stated in Table 2. This risk of infection due to annual or multiple exposures was not significant for reuse associated with the different WWTPs (*p* > 0.005).

**Table 5:** Mean probability (± 90% CI) of infection for farmers using final effluents for irrigation

	Onetime exposure	Multiple exposure
WWTP A	2.3 × 10^−4^ (± 4.7 × 10^−6^)	2.9 × 10^−2^ (± 5.7 × 10^−4^)
WWTP B	2.5 × 10^−4^ (± 5.2 × 10^−6^)	3.1 × 10^−2^ (± 6.3 × 10^−4^)
WWTP C	4.8 × 10^−4^ (± 9.9 × 10^−6^)	5.8 × 10^−2^(± 1.1 × 10^−3^)

#### Probability of *Ascaris* spp. infection for communities exposed to the treated wastewater directly and indirectly

Direct exposure to the final effluents from the centralized WWTPs either through (un)intentional immersion or swimming poses a high risk of infection. The highest mean risk of infection (2.0 × 10^−3^ (± 3.7 × 10^−5^)) was recorded with exposure to effluents from WWTP C, as was the case for the risk of infection for the farmers. Immersion in effluents from WWTP A resulted in the least probability of infection (9.6 × 10^−4^ (± 1.8 × 10^−5^)). With multiple exposures, the risk of infection increased for each of the WWTPs, where the annual risks ranged from 8.3 × 10^−2^ (± 1.4 × 10^−3^) (WWTP A) to 1.6 × 10^−1^ (± 2.5 × 10^−3^) (WWTP C) (refer to Table 6).

**Table 6:** Mean probability (± 90% CI) of infection with *Ascaris* spp. due to (un)intentional exposures to final wastewater effluents

	WWTP A		WWTP B		WWTP C	
	Onetime exposure	Annual	Onetime exposure	Annual	Onetime exposure	Annual
(Un)intentional immersion/swimming at maturation ponds or effluent contaminated surface water	9.6 × 10^−4^ (± 1.8 × 10^−5^)	8.3 × 10^−2^ (± 1.4 × 10^−3^)	1.0 × 10^−3^ (± 1.9 × 10^−5^)	9.0 × 10^−2^ (± 1.6 × 10^−3^)	2.0 × 10^−3^ (± 3.7 × 10^−5^)	1.6 × 10^−1^ (± 2.5 × 10^−3^)
Consumption of vegetables	4.1 × 10^−4^ (± 8.0 × 10^−6^)	6.1 × 10^−2^ (± 1.1 × 10^−3^)	4.5 × 10^−4^ (± 8.9 × 10^−6^)	6.6 × 10^−2^ (± 1.2 × 10^−3^)	6.9 × 10^−4^ (± 1.2 × 10^−5^)	9.7 × 10^−2^ (± 1.6 × 10^−3^)

#### Combined probability of infection for farmers exposed to treated wastewater as well as consumption of vegetables

Exposure to the effluents during irrigation and consumption of the vegetables (lettuce) from the farm annually would result in a much higher probability of infection (Table 7). Combined risks were higher in populations using effluents from WWTP C (1.0 (± 5.6 × 10^−2^)), with the least probability (7.3 × 10^−1^ (± 2.4 × 10^−2^)) with exposure to effluents from WWTP B. This difference in probability of infection was statistically significant (*p* ≤ 0.05). Based on these estimations, farmers using effluents from WWTP C who also consume their own produce are all at risk of infection with *Ascaris* spp.

**Table 7:** Mean (± 90% CI) probability of infection with *Ascaris* spp. from combined exposure to irrigation water and consumption of farm produce

Treatment plant	Probability of infection(± 90% CI)*
WWTP A	8.8 × 10^−1^ (± 8.3 × 10^−3^)
WWTP B	7.3 × 10^−1^ (± 2.4 × 10^−2^)
WWTP C	1.0 (± 5.6 × 10^−2^)

*p ≤ 0.05

## Discussion

*Ascaris* spp., hookworm, *Trichuris* spp., and *Toxocara* spp. (except in Lesotho) were the soil-transmitted helminth (STH) eggs detected in this study, including the non-STH, *Taenia* spp., with *Ascaris* spp. and hookworm the most prevalent. These are the most common helminth infections in South Africa (Appleton et al. 2009; Mkhize-Kwitshana and Mabaso 2014; Molvik et al. 2017). *Toxocara* spp. are mainly infections of animals such as dogs and cats (Chen et al. 2012; Pereira et al. 2016; Kostopoulou et al. 2017); their presence in the wastewater may therefore be from animal feces. A high prevalence and level of infection of helminths exacerbated by poor sanitation, poverty, and low water usage per capita (Chan 1997; Mara and Horan 2003) and the potentially high number of eggs excreted per day by infected individuals (10^2^–10^4^ eggs/g) (Smith and Rose 1998) all contribute to the occurrence of high concentration of helminth eggs in untreated wastewater. The variation in the concentration of these eggs in the untreated wastewater between the WWTPs is an indication of the difference in infection patterns within the cities of Durban and Maseru, mainly influenced by the factors mentioned above. Additionally, the temporal variations seen in helminth egg concentrations may be attributed to the variation in infection levels influenced by either environmental or human factors. The areas served by these treatment plants vary in terms of population size and demographic distribution. Wastewater from poor neighborhoods is expected to contain higher concentration of helminth eggs than wastewater from middle- or high-income areas (Stolk et al. 2016). Untreated wastewater at the DEWATS plant in Durban contained low concentration of these eggs, which may be attributed to the fact that this DEWATS plant treats wastewater from middle-income households. A similar trend was observed for concentrations in untreated wastewater in Lesotho, where the DEWATS plants are privately financed and treat domestic wastewater from middle- and higher-income households. A few plants were treating wastewater from schools and orphanages, and these contained higher concentrations of helminth eggs, reflecting differences in infection patterns. STH and *Taenia* spp. infections are much more prevalent in children than adults due to different exposure patterns (Anderson et al. 2015; Lo et al. 2017). The concentrations of STH and *Taenia* spp. eggs in this study are in a similar range as those from other studies in developing countries with similar socio-economic settings as South Africa and Lesotho. For instance in Brazil, Ayres (1991) reported concentrations of up to 700 eggs/L for *Ascaris* spp., 19 eggs/L for *Trichuris* spp., and 8 eggs/L for hookworm. In Tunisia, concentrations of 15 eggs/L were found (Riahi et al. 2009), and in Vietnam, 450 eggs/L were reported (Yen-Phi et al. 2010).

Performance of the WWTPs in removing STH and *Taenia* spp. eggs varied greatly but was expected to be high, due to the egg sizes, in well performing plants. Removal of *Ascaris* spp. was higher than the removal of the other helminth eggs in almost all the WWTPs with similar results reported elsewhere (Panicker and Krishnamoorthi 1978; Rose et al. 1996; Jimenez et al. 2000). The effective removal of *Ascaris* spp. eggs is probable partly due to sedimentation (Mara and Horan 2003). Eggs of *Ascaris* spp. have a specific gravity of 1.2 g/cm^3^ as compared to 1.15 g/cm^3^ and 1.055 g/cm^3^ for *Trichuris* spp. and hookworm respectively. This result in a higher settling velocity (0.77 m/h) for *Ascaris* spp. eggs than that for *Trichuris* spp. (0.73 m/h) and hookworm (0.39 m/h) (Medema et al. 1998; David and Lindquist 1982; Shuval et al. 1986; and Pike 1990). This differential terminal settling velocity based on specific gravity and other factors such as dimensions of the egg and liquid density (and temperature) are explained by Stoke’s law for discrete particle settling in sedimentation basins (Mara and Horan 2003). Additionally, the eggs may attach to particles in the wastewater aiding in their rapid sedimentation; this is most common for *Ascaris* spp. eggs (Capizzi-Banas et al. 2002; Sengupta et al. 2011). This attachment of the *Ascaris* spp. eggs to particles might have contributed to the higher removal.

In comparison, the removal of the STH and *Taenia* spp. eggs was higher in the DEWATS plants, with an average of 95–100% reduction in Lesotho, and 100% in Durban, compared to that of the centralized WWTPs where a high variation occurred. Generally, centralized WWTPs with activated sludge and trickling filter processes remove between 75 and 100% of STH eggs (Rose et al. 1996; Chaoua et al. 2017) mainly due to sedimentation. The activated sludge process has no or little effect on egg viability (Mayer and Palmer 1996; Dowd et al. 1998). In this study, the removal of viable STH eggs was recorded, resulting in lower and variable reduction figures. A high removal percentage in the DEWATS plants may be attributed to several factors. Influent wastewater is forced through the sludge bed/blanket due to the upflow baffles, whereby removal of the eggs would be enhanced by filtration and aggregation (Mara and Horan 2003). The anaerobic digestion processes (especially within the biogas digesters) may also have contributed to the inactivation of the STH eggs. Johansen et al. (2013) reported a 0.5 log reduction in viable *Ascaris suum* eggs in a mesophilic anaerobic digester at 34 °C. Hailu (2006) also reported a 50–60% reduction in STH eggs during anaerobic digestion processes from studies in Ethiopia. Additionally, the planted gravel filters (both horizontal and vertical) would further contribute to the egg removal where horizontal subsurface constructed wetlands alone have been reported to remove over 90% of STH eggs (Stott et al. 2002).

The presence of the STH and *Taenia* spp. eggs in the final effluents poses potential risk of infections. In this regard, intentional exposures, through swimming or playing nearby, pose different degrees of risk depending on the efficiency of the WWTPs. As expected, (un)intentional immersion in the final effluents from WWTP C resulted in the highest probability of infection based on the concentrations found. Exposure to the final effluents might occur in situations where the maturation ponds or final effluents are easily accessible to the community. Under such circumstances, children or even adults may swim in these and therefore exposing them to risk of infections. In other instances, workers within the WWTPs are exposed to these effluents during maintenance; for instance, it was observed in some of the WWTPs that algae and other aquatic plants grow in the ponds and therefore have to be removed, during which accidental immersion might occur exposing them to infections. Exposure to large quantities of the final effluents might not be a situation in the DEWATS plants since these are household level WWTPs and are mainly within the compounds of these houses; however, children may play close to or even within the planted gravel filters therefore exposing them to the effluents. *Ascaris* spp. eggs have a latency period of between 2 and 4 weeks, at temperatures between 15.5 and 38 °C, before they become infectious (Bogitsh et al. 2012). Therefore, the risk of infections would differ (considerably lower) from the estimates reported here. It has even been reported that at temperatures of 25 °C, *Ascaris* spp. eggs could reach the infectious stage within 10 days (Maya et al. 2012). For instance, on-site wastewater treatment systems, such as the DEWATS, increase the level of exposure to STH egg contaminated surfaces; therefore, the likelihood of infection as a result of exposure to eggs in their infective stage is enhanced and might increase the risks beyond what we reported for centralized WWTPs. In addition, the reuse of final effluents (from both the centralized WWTPs and DEWATS) for irrigation may result in the accumulation of STH eggs in the soil (Seidu et al. 2008) which allows the eggs to develop to the infective stage under the right environmental conditions, thereby also increasing the risk of infection.

WHO as part of its guidelines for safe wastewater reuse in agriculture suggested that for unrestricted agriculture, wastewater should have ≤ 1 helminth egg per liter (WHO 2006). Only final effluents from some of the DEWATS plants met the guideline. Therefore, the use of the effluents from the centralized WWTPs needs to be looked into in line with additional barriers to reduce the risks of STH infections for farmers as well as consumers of the farm produce. For instance, further treatments with storage, elevated pH, etc. may reduce the egg concentrations to safe limits (Jimenez-Cisneros and Maya-Rendon 2007).

Despite the low concentrations of STH and *Taenia* spp. eggs in the effluents from the DEWATS plants, the infection risk from the reuse of the effluents may still be higher than that of the WHO tolerable infection risk of 1 × 10^−2^ (Mara et al. 2007). This might be most likely for the few DEWATS plants where effluent quality was compromised due to system failures. The frequency and durability of such failures are determinants of the risk. It was observed in some of these plants that the biogas was not being used which led to its accumulation within the system; this reduces the hydraulic retention time which in turn reduces the treatment efficiency.

Consumption of farm produce would expose the populations to additional risk of infections, with varying probability based on the effluent quality. Except for reuse of effluents from WWTP C, the rest of the centralized WWTPs gave lower annual risk of infections as compared to the WHO tolerable risk figure for consumers. However, the combined exposure to the wastewater during irrigation and consumption of the farm produce leads to an increased risk above the tolerable risk guideline by the WHO. It was observed that wastewater reuse for irrigation was on a small scale, mainly for household consumption, whereby the possibility of a combined risk of infection due to exposure to irrigation water and consumption of the farm produce is very high (especially for the farmers). The risks of ascariasis due to exposure (either intentionally or unintentionally) to the final effluents from these WWTPs vary greatly depending on the WWTP as well as point of exposure. This variation is largely dependent on the concentration of the *Ascaris* spp. eggs in the exposure medium, which is solely dependent on the STH egg reduction efficiency of the various treatment plants and the volume/weight of exposure medium ingested.

## Conclusion

Soil-transmitted helminth and *Taenia* spp. prevalence and concentration were found to be consistent with other reports. Wastewater from low-income communities was found to be high in STH and *Taenia* spp. eggs; additionally, decentralized wastewater treatment plants located in schools and orphanages also reported high concentrations of these eggs. The link between poor communities and helminth infections needs to be studied further. The removal of STH and *Taenia* spp. eggs by the different WWTPs varied greatly depending on the type of treatment between WWTPs and also type of STH. It can be concluded that wastewater treatment achieves higher removal of *Ascaris* spp. eggs as compared to the other STH eggs reported in this study. The DEWATS plants were also found to give the highest removal efficiency of STH and *Taenia* spp. eggs as compared to the centralized WWTPs, with some of the DEWATS plants meeting the WHO guideline for wastewater reuse in irrigation. Direct or indirect exposure to effluents from these WWTPs (especially the centralized treatment plants) would therefore increase the risk of STH infections.

In conclusion, DEWATS plants in addition to their robust, cost-effective, and easy maintenance are also more efficient in removing STH eggs from wastewater, therefore making them a good option for domestic wastewater treatment, especially where effluent reuse is planned. These findings have important implications for public and environmental health protection and emerging approaches like the WHO sanitation safety planning (Hanjra et al. 2012; Winkler et al. 2017). The results obtained calls for continuous monitoring of wastewater treatment systems so as to ensure their efficiency.

## Appendix

**Table 8:** Probability distribution functions for concentrations of STH eggs in final effluents of centralized treatment plants

	*Ascaris* spp.	Hookworm	*Trichuris* spp.	*Taenia* spp.	*Toxocara* spp.
Plant A	Exponential (*λ* = 0.45455)	Exponential (*λ* = 0.26316)	Exponential (*λ* = 0.625)	Exponential (*λ* = 0.11905)	Exponential (*λ* = 0.76923)
Plant B	Exponential (*λ* = 0.41667)	Exponential (*λ* = 0.4)	Exponential (*λ* = 0.83333)	Exponential (*λ* = 0.71429)	Exponential (*λ* = 0.76923)
Plant C	Gen. Extreme Value (*k* = 0.23432; *σ* = 2.2296; *μ*= 1.8484)	Exponential (*λ* = 0.34615)	Levy (*σ* = 5.9093)	Exponential (*λ* = 0.3125)	Uniform (*a* = 2.9442; *b* = 8.9442)
